# Impact of the Clinical Trials Act on Noncommercial Clinical Research in Japan: An Interrupted Time-series Analysis

**DOI:** 10.2188/jea.JE20210051

**Published:** 2022-01-05

**Authors:** Ikuyo Tsutsumi, Yusuke Tsutsumi, Chikashi Yoshida, Takuya Komeno, Yuichi Imanaka

**Affiliations:** 1 Department of Healthcare Economics and Quality Management, Kyoto University Graduate School of Medicine, Kyoto, Japan; 2 Department of Hematology, National Hospital Organization Mito Medical Center, Ibaraki, Japan; 3 Department of Emergency Medicine, National Hospital Organization Mito Medical Center, Ibaraki, Japan; 4 Department of Healthcare Epidemiology, Kyoto University Graduate School of Medicine, Kyoto, Japan

**Keywords:** Clinical Trials Act, interrupted time-series analysis, clinical research

## Abstract

**Background:**

The number of new noncommercial clinical studies conducted in Japan declined within the first year of the implementation of the Clinical Trials Act (CTA) on April 1, 2018. This study aimed to examine the impact of the CTA’s enforcement on the number of new noncommercial clinical studies registered in the Japanese Clinical Trial Registry.

**Methods:**

An interrupted time-series design was used in the analysis, which was conducted from April 2015 to March 2019. We collected data for studies registered in the Clinical Trial Registry, managed by the University Hospital Medical Information Network.

**Results:**

In total, 35,811 studies were registered; of these, 16,455 fulfilled the eligibility criteria. The difference in the trend of monthly number of new studies after CTA enforcement decreased significantly by 15.0 (95% confidence interval [CI], −18.7 to −11.3), and the level decreased by 40.8 (95% CI, −68.2 to −13.3) studies from the pre-enforcement to the post-enforcement period. Multigroup analyses indicated that the act exerted a significant effect on the trend of new clinical studies, particularly those with smaller sample sizes, interventional study designs, and nonprofit funding sponsors.

**Conclusions:**

The number of Japanese noncommercial clinical studies declined significantly following implementation of the CTA. It is necessary to establish a system to promote clinical studies in Japan while ensuring transparency and safety.

## INTRODUCTION

Recently, various regulations and laws have been developed for clinical research worldwide because research misconduct and inappropriate relationships between pharmaceutical companies and researchers have become serious problems. Such “research scandals” have recently become important ethical issues in Japan as well. It was exposed that data falsification and conflicts of interest occurred in research conducted in various fields, resulting in papers being withdrawn in several clinical studies and serious confusion in clinical practice.^1^ Research misconduct can impair data accuracy and cause disadvantages to research participants and those who would benefit from the results.

Commercial clinical trials, conducted to obtain national marketing approval for drugs and medical devices, are legally regulated by the International Conference on Harmonization Good Clinical Practice Guideline in Japan. Meanwhile, noncommercial clinical studies, including non-commercial interventional studies and non-interventional studies, are regulated by the Ethical Guidelines for Medical and Health Research Involving Human Subjects, which are not legally enforceable.

To improve the conduct of clinical studies by ensuring trust in clinical studies and thereby promote public health and hygiene, the Clinical Trials Act (CTA) was established by the Ministry of Health, Labour and Welfare (MHLW) in Japan and enforced since April 1, 2018.^2^ It aimed to define procedures for the conduct of clinical studies, appropriate provision of the management of reviews by certified review boards (CRBs), and systems for disclosure of information regarding funding or other benefits for clinical studies.^3^ The CTA mainly regulates the “specified clinical trials” which are defined as the noncommercial interventional studies receiving funds or benefits from manufacturers and that using unapproved/off-label use drugs/medical devices. The Act also covers noncommercial interventional studies other than “specified clinical trials”, which is not mandatory, but recommended (duty of effort). The commercial trials that are conducted to obtain national marketing approval for drugs and medical devices are exempted from the CTA. The CTA requires 1) contracts and disclosure of information regarding research funding; 2) review of the implementation plan and adverse events by a CRB authorized by the MHLW; 3) compliance with implementation standards for monitoring, conflict of interest management, and record preservation; and 4) disclosure of the implementation plan to the MHLW.

While legal regulations for clinical studies have been implemented in various countries to ensure transparency, overregulation can sometimes limit clinical studies, particularly those of a noncommercial nature.^5^^,^^6^ In the European Union (EU), the number of studies submitted for research grants or ethical review has declined by 30% to 50%, while the proportion of noncommercial studies has decreased from 40% to 14% since Directive 2001/20/EC was adopted in April 2001 and launched in May 2004.^7^^,^^8^ Similarly, in Japan, there is the concern that the new law could reduce the number of studies conducted at study institutes lacking financial support, but this has not been examined previously. This study aimed to clarify the association between the enforcement of the CTA and the number of studies newly registered in the Japanese Clinical Trial Registry, using an interrupted time-series analysis design.

## METHODS

### Data source

We collected data from the Clinical Trials Registry managed by the University hospital Medical Information Network Clinical Trials Registry (UMIN-CTR),^9^ which is part of the Japan Primary Registries Network (JPRN). The JPRN consists of the following three clinical study registration agencies: UMIN-CTR; Japan Pharmaceutical Information Center Clinical Trial Information^10^; and the Clinical Trials Registry operated by the Center for Clinical Trials of the Japan Medical Association.^11^ The JPRN is a clinical trial registry that meets the criteria of the International Committee of Medical Journal Editors and was authorized by the World Health Organization (WHO) Primary Registry on August 16, 2008.^12^ UMIN-CTR welcomes the registration of all academic clinical studies, including both commercial and noncommercial clinical trials, and both interventional and noninterventional studies. However, in practice, pharmaceutical company-led commercial clinical trials are registered in the Japan Pharmaceutical Information Center Clinical Trial Information, and physician-led commercial and medical device clinical trials are registered at the Center for Clinical Trials, Japan Medical Association. Additionally, the newly established clinical study database, the Japan Registry of Clinical Trials (jRCT), was added to the JPRN and approved by the WHO Primary Registry on December 5, 2018. The UMIN-CTR provides open .csv files, including daily snapshots of studies registered in the database. We downloaded the file from the UMIN-CTR website on April 1, 2019.^9^

### Outcomes

The primary outcome was the change in the trend, defined as the difference in changes (slope) in the monthly number of new clinical studies before and after the enforcement of the CTA. The secondary outcome was the change in the monthly number of new clinical studies, defined as the difference in the monthly number of new clinical studies from the end of the pre-CTA period to the period immediately following the enforcement of the CTA. Additionally, the study focused on differences in the effects of the CTA on the following factors: sample size (≤100 or >100), study objectives (malignancy or nonmalignancy), study design (interventional or noninterventional), and type of funding sponsor (for-profit or nonprofit).

### Data selection

The inclusion criterion was an anticipated study start date between April 1, 2015, and March 31, 2019. The exclusion criteria were the exclusion of Japan from the study region and commercial trials requiring Investigational New Drug applications to the MHLW, because these trials were applicable to the International Conference on Harmonization Good Clinical Practice Guideline rather than the CTA. We included noninterventional studies, such as observational studies and meta-analyses, in this study since one of the study objectives was to determine whether the number of regulated interventional studies had been more affected by the Clinical Trials Act compared to unregulated designs.

### Statistical analysis

Descriptive analyses were performed based on the baseline characteristics of studies before and after the enforcement of the CTA. Continuous variables are presented as medians (interquartile ranges [IQRs]), and categorical variables are presented as frequencies and percentages. A Wilcoxon rank-sum test was performed to compare continuous variables, and Pearson’s chi-squared test was used in between-group comparisons of categorical or binary variables. An interrupted time-series analysis (ITSA) design^13^^,^^14^ was used to assess the association between the enforcement of the CTA and changes in the trends and the monthly number of new studies. The intervention of interest was the enforcement of the CTA. The first month of the intervention period was set as April 2018, and the analysis period lasted for 48 months, from April 2015 to March 2019. To create the time-series dataset, the aggregate number of studies for each month from April 2015 to March 2019 was tabulated according to the anticipated study start date. We performed ITSA using two ordinary least-squares regression-based approaches.^15^

The following regression equation was used in the single-group analysis^15^^–^^18^:

$$Y_{t}=\beta_{0}+\beta_{1}T_{t}+\beta_{2}X_{t}+\beta_{3}X_{t}T_{t}+\epsilon_{t}$$

The following regression equation was used in the multigroup analysis^14^^–^^17^:

$$\begin{array}{rl} Y_{t} & =\beta_{0}+\beta_{1}T_{t}+\beta_{2}X_{t}+\beta_{3}X_{t}T_{t}+\beta_{4}Z\\ & \;+\beta_{5}ZT_{t}+\beta_{6}ZX_{t}+\beta_{7}ZX_{t}T_{t}+\epsilon_{t} \end{array}$$

*Y_t_* represents the monthly number of studies measured at time point *t*, and *T_t_* represents the time since April 2015. *X_t_* is a dummy variable representing the enforcement of the CTA (eg, pre-CTA period was 0, otherwise 1). In the single-group analysis, β_0_ represents the number of studies in the first month of the study (ie, April 2015). β_1_ represents the slope of the monthly number of studies (trend) before the implementation of the CTA. β_2_ represents the change in the monthly number of studies from the end of the pre-CTA period (level change) to the period immediately following the enforcement of the CTA. β_3_ indicates the slope change following the enforcement of the CTA. In the multigroup analysis, β_0_ to β_3_ represents the value for the control group, and β_4_ to β_7_ represents the value for the comparison group. β_4_ represents the difference in the number of studies during the first month of the study between the control group and comparison group (difference in level) prior to enforcement of the CTA. β_5_ represents the difference in the slopes of the monthly number of studies between the control group and comparison group (difference in trend) prior to enforcement of the CTA. β_6_ represents the difference in levels immediately following enforcement of the CTA between the control group and comparison group. β_7_ represents the difference in slopes (trends) in the pre- and post-CTA periods between the control group and comparison group. Calendar month was included as a dummy variable, to account for seasonality. Newey-West standard errors were used to deal with autocorrelation and possible heteroskedasticity.^15^ All statistical tests were two-sided, and the significance level was set at 5%. All analyses were performed using Stata SE version 14.2 (Stata Corp, College Station, TX, USA). The requirement for ethics committee approval was waived because all the data are publicly available online and comprise only aggregate values, without any personally identifiable information.

## RESULTS

**Figure 1.**
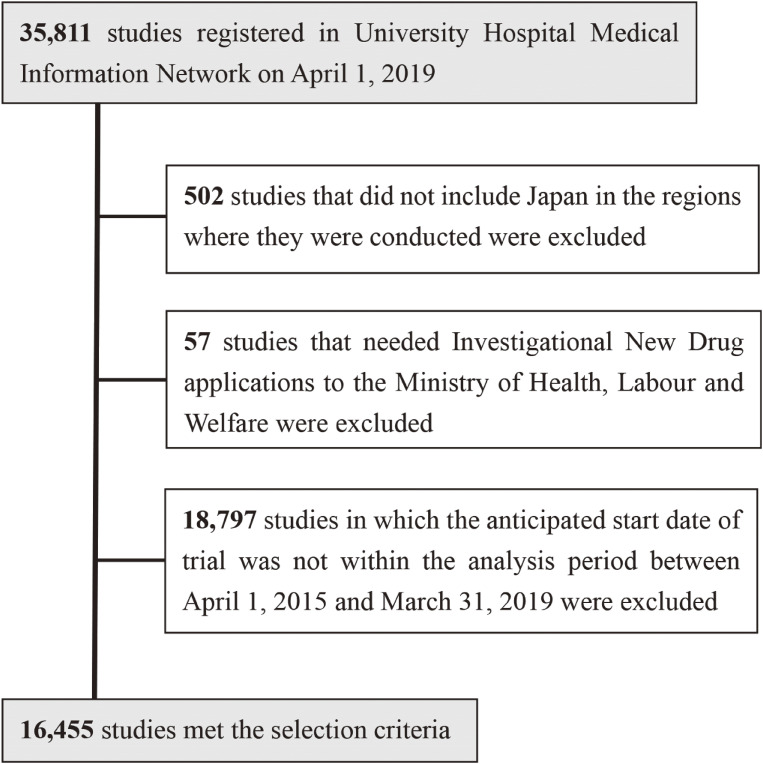
Flow diagram of data selection. MHLW, Ministry of Health, Labour and Welfare; UMIN-CTR, University hospital Medical Information Network.

Figure 1 shows the flow chart for data selection. We downloaded all data for 35,811 studies in the UMIN-CTR on April 1, 2019. The 502 studies that were not conducted in Japan and the 57 that involved Investigational New Drug applications to the MHLW were excluded according to the exclusion criteria. Additionally, 18,797 studies in which the anticipated study start date was not between April 1, 2015, and March 31, 2019, were excluded from the analysis. Therefore, 16,455 studies ultimately met the selection criteria.

Table 1 shows the baseline characteristics of studies initiated between April 2015 and March 2019. The proportion of interventional studies conducted after the enforcement of the CTA was lower relative to that of those conducted before the enforcement of the CTA (from 70.8% to 66.6%, *P* < 0.001). Regarding disease classification, the proportion of studies involving internal medicine and surgery conducted after the enforcement of the CTA was lower relative to that of those conducted before (from 43.0% to 40.0% and from 12.6% to 9.3%, respectively; *P* = 0.002). Moreover, the proportion of studies involving malignancy decreased after the law was enforced (from 24.4% to 21.7%, *P* < 0.001). Conversely, the proportion of studies involving healthy people increased after the enforcement (from 22.6% to 29.0%, *P* < 0.001). Regarding types of funding organizations, the most common was self-funded, followed by for-profit organizations and Japanese governmental offices, both before and after the enforcement of the CTA.

**Table 1.** Baseline characteristics of studies that began between April 2015 and March 2019

	Total *N* = 16,455	Before the Act *n* = 13,095	After the Act *n* = 3,360	*P* value
**Study design,** *N* (%)				
Interventional	11,506 (69.9%)	9,268 (70.8%)	2,238 (66.6%)	<0.001^***^
Observational	4,656 (28.3%)	3,574 (27.3%)	1,082 (32.2%)	
Other (eg, meta-analysis)	248 (1.5%)	208 (1.6%)	40 (1.2%)	
Not selected	45 (0.3%)	45 (0.3%)	0 (0.0%)	
**Sample size,** median (IQR)^a^	50 (25–110)	50 (25–110)	50 (24–116.5)	0.97
**Category by sample size,** *N* (%)^a^				
≤100	12,261 (74.6%)	9,769 (74.7%)	2,492 (74.2%)	0.52
>100	4,175 (25.4%)	3,307 (25.3%)	868 (25.8%)	
**Basic objectives (primary outcome),** *N* (%)				
Safety	1,238 (7.5%)	977 (7.5%)	261 (7.8%)	<0.001^***^
Efficacy	7,165 (43.5%)	5,622 (42.9%)	1,543 (45.9%)	
Safety and efficacy	4,413 (26.8%)	3,650 (27.9%)	763 (22.7%)	
Bioequivalence	171 (1.0%)	136 (1.0%)	35 (1.0%)	
Bioavailability	190 (1.2%)	158 (1.2%)	32 (1.0%)	
Pharmacokinetics	143 (0.9%)	117 (0.9%)	26 (0.8%)	
Pharmacodynamics	48 (0.3%)	46 (0.4%)	2 (0.1%)	
Pharmacokinetics and pharmacodynamics	79 (0.5%)	63 (0.5%)	16 (0.5%)	
Other	3,008 (18.3%)	2,326 (17.8%)	682 (20.3%)	
**Disease classification by specialty,** *N* (%)				
Internal medicine	6,971 (42.4%)	5,628 (43.0%)	1,343 (40.0%)	0.002^**^
Surgery	1,961 (11.9%)	1,649 (12.6%)	312 (9.3%)	<0.001^***^
Medicine, other	6,814 (41.4%)	5,498 (42.0%)	1,316 (39.2%)	0.003^**^
Dental medicine	491 (3.0%)	384 (2.9%)	107 (3.2%)	0.44
Nursing	416 (2.5%)	317 (2.4%)	99 (2.9%)	0.083
Healthy people	3,926 (23.9%)	2,953 (22.6%)	973 (29.0%)	<0.001^***^
Not applicable	887 (5.4%)	661 (5.0%)	226 (6.7%)	<0.001^***^
**Disease classification by malignancy,** *N* (%)				
Malignancy	3,927 (23.9%)	3,198 (24.4%)	729 (21.7%)	<0.001^***^
Other	12,528 (76.1%)	9,897 (75.6%)	2,631 (78.3%)	
**Category of funding organization,** *N* (%)				
For-profit organization	3,186 (19.4%)	2,491 (19.0%)	695 (20.7%)	<0.001^***^
Self-funding	5,388 (32.7%)	4,407 (33.7%)	981 (29.2%)	
Japanese governmental office	1,995 (12.1%)	1,518 (11.6%)	477 (14.2%)	
Nonprofit foundation	453 (2.8%)	357 (2.7%)	96 (2.9%)	
Local government	282 (1.7%)	234 (1.8%)	48 (1.4%)	
Government offices of other countries	161 (1.0%)	121 (0.9%)	40 (1.2%)	
Outside Japan	51 (0.3%)	38 (0.3%)	13 (0.4%)	
Other	4,939 (30.0%)	3,929 (30.0%)	1,010 (30.1%)	

IQR, interquartile range.

^a^Data for 19 of 13,095 studies were missing in the sample before the act’s enforcement.

^*^P < 0.05; ^**^P < 0.01; ^***^P < 0.001.

**Figure 2.**
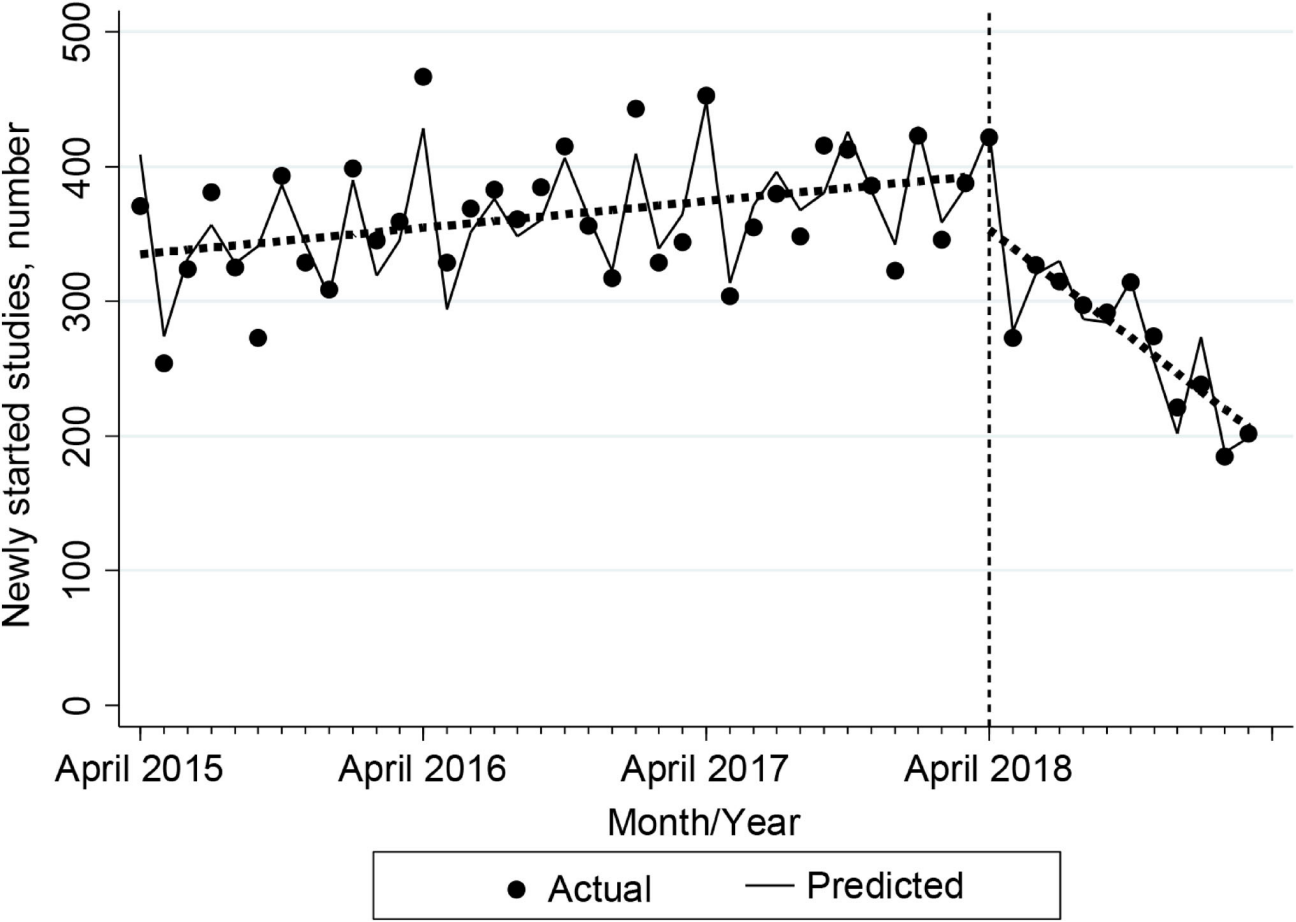
Results of the single-group ITSA for monthly number of new studies. The points on the figure represent the actual monthly number of studies. The solid lines indicate the predicted monthly number of studies adjusted by calendar month. Dotted lines represent trends (slopes) in monthly numbers of studies. Level change after the enforcement of the Clinical Trials Act (CTA) is defined as the difference from the end of the dotted line during the pre-act period to the starting point of the dotted line during the post-act period. CI, confidence interval; ITSA, interrupted time-series analysis.

Figure 2 and Table 2 shows the results of the single-group ITSA for the monthly number of new studies. During the pre-enforcement period, the trend in the number of new studies increased by 1.64 (95% CI, 0.71–2.57, *P* = 0.001) each month. In contrast, during the post-CTA period, this trend was expected to decline significantly, by 13.3 (95% CI, −17.1 to −9.63, *P* < 0.001) studies every month. The difference in trends before and after the enforcement of the CTA was −15.0 (95% CI, −18.7 to −11.3, *P* < 0.001) studies. Furthermore, there was a significant decline in levels (−40.8; 95% CI, −68.2 to −13.3, *P* = 0.005) after the enforcement.

**Table 2.** Results of the single-group ITSA for monthly number of new studies

	β	95% CI			*P*
Trend before the enforcement of the CTA :*β*_1_	1.64	0.71	–	2.57	0.001^**^
Trend after the enforcement of the CTA :*β*_1_+*β*_3_	−13.3	−17.1	–	−9.63	<0.001^***^
Difference in trend before and after the enforcement of the CTA :*β*_3_	−15.0	−18.7	–	−11.3	<0.001^***^
Difference in level before and after the enforcement of the CTA :*β*_2_	−40.8	−68.2	–	−13.3	0.005^**^

CI, confidence interval; CTA, Clinical Trials Act.

^*^P < 0.05; ^**^P < 0.01; ^***^P < 0.001.

**Figure 3.**
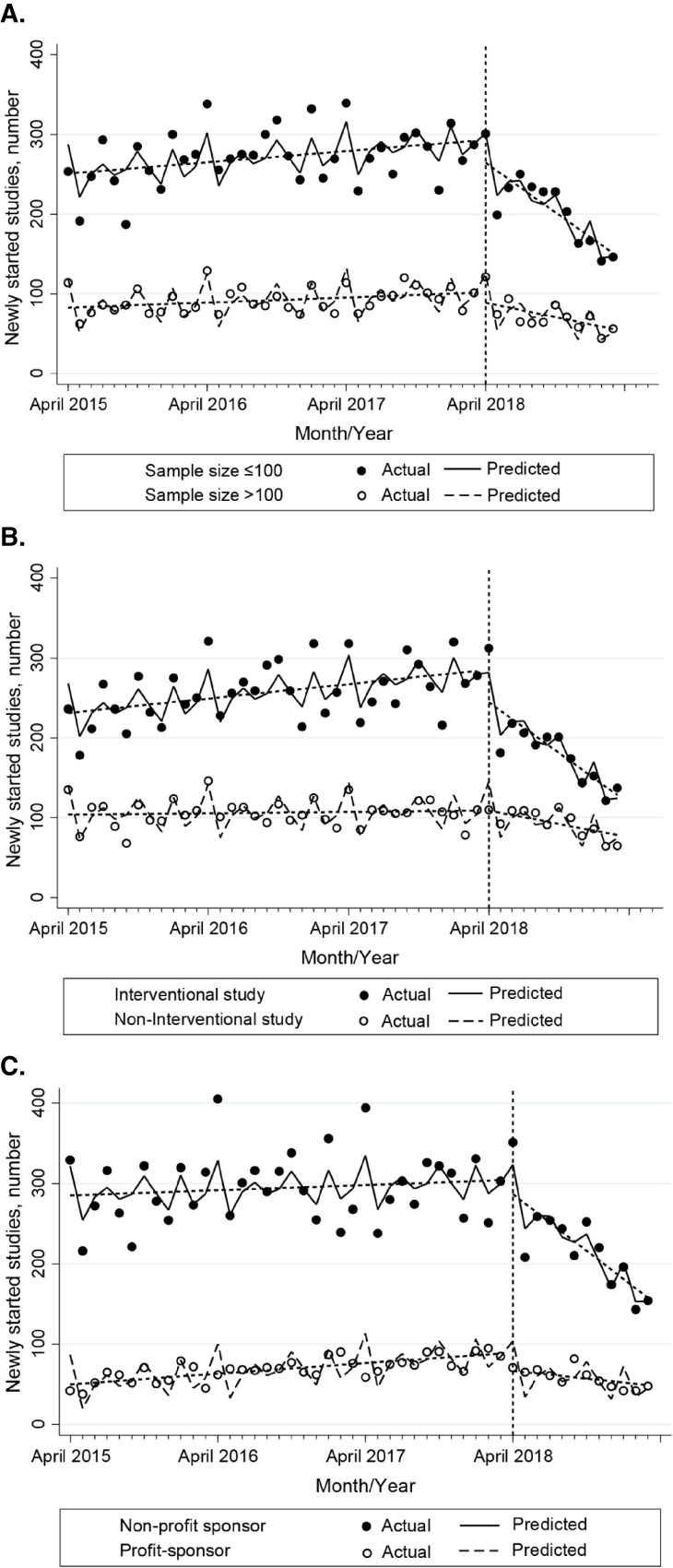
Results of the multigroup ITSA for monthly number of new studies. (**A**) ITSA according to sample size. (**B**) ITSA according to study design. (**C**) ITSA according to type of funding sponsor. The points on the graph represent the actual monthly number of studies. Solid lines indicate the predicted monthly number of studies adjusted by calendar month. Dotted lines represent trends (slopes) in monthly number of studies. Level change after the enforcement of the Clinical Trials Act (CTA) is defined as the difference from the end of the dotted line during pre-act period to the starting point of the dotted line during post-act period. CI, confidence interval; ITSA, interrupted time-series analysis.

Figure 3 and Table 3 represent the results of the multigroup ITSA examining the effects of the CTA on various factors such as sample size, study design, and type of funding sponsor. As shown in Figure 3A, the pre-CTA trend for studies with sample sizes >100 increased by 0.53 (95% CI, 0.22–0.84, *P* = 0.001) monthly, which did not show a significant difference from those of studies with sample sizes ≤100 (0.65; 95% CI, −0.29 to 1.58, *P* = 0.17). In contrast, the post-CTA trend for studies with sample sizes >100 decreased by 2.99 (95% CI, −5.33 to −0.65, *P* = 0.013) monthly, while studies with sample sizes ≤100 showed a significant difference from those of studies with sample sizes >100 (−7.33; 95% CI, −10.6 to −4.11, *P* < 0.001). There was no significant difference in level changes between studies with sample sizes ≤100 and >100 during the period immediately following the enforcement of the CTA (−15.8; 95% CI, −45.4 to 13.8, *P* = 0.29).

**Table 3.** Results of the multigroup ITSA for monthly number of new studies

	Sample size ≤100 vs Sample size >100					Interventional studies vs Non-interventional studies					Non-profit sponsor vs Profit sponsor				
		
	β	95% CI			*P*	β	95% CI			*P*	β	95% CI			*P*
Pre-CTA trend for studies with sample sizes >100 :*β*_1_	0.53	0.22	–	0.84	0.001^**^	0.14	−0.24	–	0.52	0.47	1.10	0.68	–	1.52	<0.001^***^
Post-CTA trend for studies with sample sizes >100 :*β*_1_+*β*_3_	−2.99	−5.33	–	−0.65	0.013^*^	−2.78	−5.21	–	−0.35	0.026^*^	−1.61	−3.99	–	0.77	0.18
Difference in trend for studies with sample sizes >100 before and after the enforcement of the CTA :*β*_3_	−3.52	−5.80	–	−1.24	0.003^**^	−2.92	−5.40	–	−0.44	0.021^*^	−2.71	−4.89	–	−0.53	0.016^*^
Difference in level for studies with sample sizes >100 before and after the enforcement of the CTA :*β*_2_	−12.9	−33.8	–	8.01	0.22	−0.08	−16.9	–	16.7	0.99	−22.7	−42.4	–	−3.12	0.024^*^
Difference in Pre-CTA trend between studies with sample sizes ≤100 and those >100 :*β*_5_	0.65	−0.29	–	1.58	0.17	1.36	0.54	–	2.19	0.001^**^	−0.55	−1.44		0.34	0.22
Difference in Post-CTA trend between studies with sample sizes ≤100 and those >100 :*β*_5_+*β*_7_	−7.33	−10.6	–	−4.11	<0.001^***^	−7.78	−10.5	–	−5.05	<0.001^***^	−10.1	−12.0		−8.27	<0.001^***^
Difference in trend before and after the enforcement of the CTA between studies with sample sizes ≤100 and those >100 :*β*_7_	−7.97	−11.3	–	−4.62	<0.001^***^	−9.14	−12.0	–	−6.31	<0.001^***^	−9.56	−11.6		−7.52	<0.001^***^
Difference in level before and after the enforcement of the CTA between studies with sample sizes ≤100 and those >100 :*β*_6_	−15.8	−45.4	–	13.8	0.29	−40.6	−65.0	–	−16.2	0.001^**^	4.71	−14.6		24.0	0.63

CI, confidence interval; CTA, Clinical Trials Act.

^*^P < 0.05; ^**^P < 0.01; ^***^P < 0.001.

Moreover, Figure 3B shows the effects of the CTA according to study design. The pre-CTA trend in noninterventional studies did not increase significantly (0.14; 95% CI, −0.24 to 0.52, *P* = 0.47), while interventional studies increased significantly by 1.36 (95% CI, 0.54–2.19, *P* = 0.001) per month. The post-CTA trend for noninterventional studies decreased by 2.78 (95% CI, −5.21 to −0.35, *P* = 0.026) per month, while interventional studies showed a significant difference from that for noninterventional studies (−7.78; 95% CI, −10.5 to −5.05, *P* < 0.001). There was a significant difference in level changes between interventional and noninterventional studies during the period immediately following the enforcement of the CTA (−40.6; 95% CI, −65.0 to −16.2, *P* = 0.001).

Furthermore, Figure 3C represents the results of the ITSA according to the type of funding sponsor. The pre-CTA trend for studies funded by for-profit sponsors increased at a rate of 1.10 (95% CI, 0.68–1.52, *P* < 0.001) studies per month, which did not differ significantly from studies funded by nonprofit sponsors (−0.55; 95% CI, −1.44 to 0.34, *P* = 0.22). In contrast, the post-CTA trend for studies funded by for-profit sponsors decreased at a rate of 1.61 (95% CI, −3.99 to 0.77, *P* = 0.18) studies per month, while studies funded by nonprofit sponsors showed a significant difference from that for studies funded by for-profit sponsors (−10.1; 95% CI, −12.0 to −8.27, *P* < 0.001). There was no significant difference in level changes between studies funded by for- and nonprofit sponsors during the period immediately following the enforcement of the CTA (4.71; 95% CI, −14.6 to 24.0, *P* = 0.63).

## DISCUSSION

The results of the single-group ITSA showed that the total number of new studies declined significantly in both trends and levels following the enforcement of the CTA in April 2018. The current data indicated that the enforcement of the CTA exerted a strong negative effect on the number of new clinical studies. The analysis of various factors, using the multigroup ITSA method, showed that the trend decreased significantly for all types of studies after the new legal regulation, especially those with smaller sample sizes, interventional study designs, and nonprofit funding sponsors. The result suggests that enforcement of the CTA particularly affected studies with limited human resources and financial support. Prior to implementation, there were no legal restrictions in Japan on noncommercial clinical studies. After introduction of the new law, CRBs authorized by the MHLW reviewed study plans, adverse event reports, and the exact status of conflicts of interest of all physicians involved in the studies.^2^^–^^4^ These improvements seek to increase the transparency of the procedures involved in clinical studies and flow of expenses, which contribute to the prevention of research misconduct. However, substantial research funding is needed to disburse expensive commission fees for review by a CRB and management costs, including personnel expenses for following requirements mandated by the CTA, as compared to the period prior to its introduction. Therefore, it can be difficult for researchers who do not have sufficient human resources and funds to conduct new clinical research.

Before the CTA was enacted in 2018, new ethical guidelines were established in 2015; the Act on the Protection of Personal Information was revised in 2015 and enacted in 2017. Therefore, it was necessary to confirm whether the decline in new research in the past few years was due to the CTA and not these factors. In this study, we used ITSA to show that there was a significant decrease in new clinical studies before and after the CTA was implemented. We also conducted analyses using ITSA, before and after the implementation of the new ethical guidelines and the revised Act on the Protection of Personal Information, and confirmed that there was no significant decrease in the number of new clinical studies after the implementation of each (data not shown).

Since 2001, each country in the EU has developed its own directives for clinical studies to maintain the quality of studies based on Directive 2001/20/EC. Thus, in most countries, the number of noncommercial studies has decreased, particularly those funded by nonprofit sponsors,^19^^–^^21^ because regulatory bodies impose highly demanding stipulations and expensive fees for the submission of studies to ethics committees. There are similar concerns in Japan that the CTA may also overly regulate clinical studies regardless of the study subject and magnitude of the risk to participants. Only a limited number of organizations with an accessible labor force and adequate financial resources could conduct clinical studies with larger sample sizes, which may limit the scopes of study domains. Indeed, a Japanese questionnaire survey showed that the investigators’ desire for support systems when conducting clinical studies was significantly higher after the implementation of the CTA than before.^21^ Furthermore, it may cause a decline in the number of new researchers who conduct innovative clinical research. It is essential to develop a system in which physicians can obtain appropriate support to conduct research. We may be able to follow the model developed in Italy, where the number of clinical studies increased after the introduction of legal regulation.^21^ This is apparently because Italy implemented policy changes that include waiving of ethical review fees, prompt approval by ethics review boards, financial support for research expenses or management, and alleviation of regulations on investigator-driven study for noncommercial research.^22^^–^^24^

This study was subject to a few limitations. First, it did not include jRCT data analyzed in this study. Although the jRCT was enforced in April 2018, the actual registration begun in April 2019. In addition, there were only six new studies registered in the jRCT, but not in UMIN-CTR, within the current analysis period between April 1, 2015, and March 31, 2019, as reported in the WHO International Clinical Trials Registry Platform. This suggests that exclusion of the jRCT data has little impact on the current results. Second, we could analyze the data for only 1 year after the enforcement of the CTA. It may be essential to carry out medium- and long-term analysis for examining the impact of the CTA on clinical research in Japan. Nevertheless, the current analysis contributes to identifying the key issues faced by Japanese clinical research under the new legal regulation, which can be used to address them quickly. Finally, it is difficult to assess whether the introduction of the CTA can in fact reduce clinical research with inadequate transparency and reliability, which is the original purpose of the legislation, despite the decrease in newly initiated research found in this study. Further study from an alternative perspective may be needed to clarify this issue.

In conclusion, the number of noncommercial clinical studies decreased 1 year after the implementation of the CTA in Japan. Establishing a new system to promote clinical research in Japan while ensuring research transparency and safety is vital.

## References

[1] Sawano T, Ozaki A, Saito H, Shimada Y, Tanimoto T. Payments from pharmaceutical companies to authors involved in the Valsartan scandal in Japan. JAMA Netw Open. 2019;2(5):e193817. 10.1001/jamanetworkopen.2019.3817 31099864 PMC6537813

[2] Ministry of Health, Labour and Welfare. About the Clinical Trials Act. https://www.mhlw.go.jp/stf/seisakunitsuite/bunya/0000163417.html; 2019 Accessed 12.12.2019. [Japanese].

[3] Ministry of Health, Labour and Welfare. Clinical Trials Act (Act No. 16 of April 14, 2017). 2017. https://www.mhlw.go.jp/file/06-Seisakujouhou-10800000-Iseikyoku/0000213334.pdf; 2019 Accessed 12.12.2019. [Japanese].

[4] Tashiro S. How the clinical research act will change clinical research in Japan. Gan To Kagaku Ryoho. 2018;45(7):1011–1016. 30042262

[5] Morice AH. The death of academic clinical trials. Lancet. 2003;361(9368):1568. 10.1016/S0140-6736(03)13211-4 12737908

[6] The Lancet. Who’s afraid of the European Clinical Trials Directive? Lancet. 2003;361(9376):2167. 10.1016/S0140-6736(03)13781-6 12842362

[7] Bosch X. Europe’s restrictive rules strangling clinical research. Nat Med. 2005;11(12):1260. 10.1038/nm1205-1260b16333252

[8] McMahon AD, Conway DI, Macdonald TM, McInnes GT. The unintended consequences of clinical trials regulations. PLoS Med. 2009;3(11):e1000131. 10.1371/journal.pmed.1000131 19918557 PMC2768793

[9] University hospital Medical Information Network (UMIN). UMIN Clinical Trials Registry (UMIN-CTR). https://www.umin.ac.jp/ctr/index.htm; 2013 Accessed 17.06.2020.

[10] Japan Pharmaceutical Information Center. Japic Clinical Trials Information. https://www.clinicaltrials.jp/cti-user/common/Top.jsp; 2019 Accessed 12.08.2019. [Japanese].

[11] The Japan Medical Association Center for Clinical Trials. JMACCT Clinical Trials Registry. https://dbcentre3.jmacct.med.or.jp/JMACTR/Default_Eng.aspx; 2019 Accessed 12.08.2019.

[12] World Health Organization. International Clinical Trials Registry Platform (ICTRP). https://www.who.int/ictrp/network/primary/en/; 2019 Accessed 11.09.2019.

[13] Wagner AK, Soumerai SB, Zhang F, Ross-Degnan D. Segmented regression analysis of interrupted time series studies in medication use research. J Clin Pharm Ther. 2002;27(4):299–309. 10.1046/j.1365-2710.2002.00430.x 12174032

[14] Bernal JL, Cummins S, Gasparrini A. Interrupted time series regression for the evaluation of public health interventions: a tutorial. Int J Epidemiol. 2017;46(1):348–355. 10.1093/ije/dyw098 27283160 PMC5407170

[15] Linden A. Conducting interrupted time-series analysis for single- and multiple-group comparisons. Stata J. 2015;15(2):480–500. 10.1177/1536867X1501500208

[16] Linden A, Adams JL. Applying a propensity score-based weighting model to interrupted time series data: improving causal inference in programme evaluation. J Eval Clin Pract. 2011;17(6):1231–1238. 10.1111/j.1365-2753.2010.01504.x 20973870

[17] Simonton DK. Cross-sectional time-series experiments: some suggested statistical analyses. Psychol Bull. 1977;84(3):489–502. 10.1037/0033-2909.84.3.489

[18] Simonton DK. Erratum to Simonton. Psychol Bull. 1977;84(6):1097. 10.1037/h0078052

[19] Frewer LJ, Coles D, Champion K, . Has the European clinical trials directive been a success? BMJ. 2010;340:c1862. 10.1136/bmj.c1862 20382668

[20] Hartmann M, Hartmann-Vareilles F. The clinical trials directive: how is it affecting Europe’s noncommercial research? PLoS Clin Trials. 2006;1(2):e13. 10.1371/journal.pctr.0010013 16871335 PMC1488897

[21] Chuma M, Takechi K, Yagi K, . Academic investigators’ interest in promoting specified clinical trials: questionnaire survey before and after implementation of the Clinical Trial Act. J Med Invest. 2021;68(1.2):71–75. 10.2152/jmi.68.71 33994483

[22] Hartmann M. Impact assessment of the European clinical trials directive: a longitudinal, prospective, observational study analyzing patterns and trends in clinical drug trial applications submitted since 2001 to regulatory agencies in six EU countries. Trials. 2012;13:53. 10.1186/1745-6215-13-53 22540886 PMC3349611

[23] Filibeck U, Addis A, Tomino C, Martini N. European clinical trials directive: the Italian position. Lancet. 2004;363(9421):1651–1652. 10.1016/S0140-6736(04)16222-3 15145650

[24] Gazzetta Ufficiale. GU Serie Generale n.43 del 22-02-2005. https://www.gazzettaufficiale.it/eli/gu/2005/02/22/43/sg/pdf 2019; 2019 Accessed 12.11.2019. [Italian].

